# GnRH Agonist with Low-Dose hCG (Dual Trigger) versus GnRH Agonist Only Trigger in High Responders in GnRH Antagonist Cycle: A Systematic Review and Meta-Analysis

**DOI:** 10.1159/goi/aczag007

**Published:** 2026-08-26

**Authors:** Hanom Husni Syam, Hartanto Bayuaji, Dian Tjahyadi, Anita Rachmawati, Mulyanusa Amarullah Ritonga, Nicholas Adrianto

**Affiliations:** a Division of Reproductive Endocrinology and Infertility, Department of Obstetrics and Gynecology, Padjadjaran University, Dr. Hasan Sadikin General Hospital, Bandung, Indonesia; b School of Medicine and Health Sciences, Atma Jaya Catholic University of Indonesia, Jakarta, Indonesia

**Keywords:** Gonadotropin-releasing hormone agonist, Human chorionic gonadotropin, High ovarian response, Fertilization in vitro, Ovarian hyperstimulation syndrome, Infertility

## Abstract

**Introduction:**

Ovarian hyperstimulation syndrome (OHSS) remains a serious complication of assisted reproductive technology, particularly in high ovarian responders. Although a GnRHa-only trigger effectively minimizes the risk of OHSS, it has been associated with reduced oocyte yield and, in some reports, lower oocyte maturity, and may also compromise luteal function. Dual trigger, combining GnRHa with low-dose human chorionic gonadotropin (hCG), was developed to enhance reproductive outcomes; however, concerns persist that even low-dose hCG may reintroduce OHSS risk. This systematic review and meta-analysis aimed to compare the efficacy and safety of dual trigger versus GnRHa-only trigger in high ovarian responders.

**Methods:**

A systematic search of PubMed, Scopus, and Google Scholar from January 2000 to December 2025 identified studies comparing dual trigger (GnRHa plus low-dose hCG) with GnRHa-only trigger in high responders undergoing IVF/ICSI. Randomized and observational cohort studies reporting reproductive outcomes or moderate-to-severe OHSS were included. Risk ratios (RRs) were calculated for reproductive outcomes, and odds ratios (ORs) were calculated for OHSS using random-effects models. Risk of bias was assessed using ROBINS-I, and the certainty of the evidence was evaluated using the GRADE framework.

**Results:**

Five retrospective cohort studies were included. Dual trigger did not improve the number of oocytes retrieved, the mature oocyte rate, the fertilization rate, the embryo quality, the clinical pregnancy rate, the live birth rate, the cumulative live birth rate, or the miscarriage rate compared with the GnRHa-only trigger. In contrast, dual trigger was associated with increased risk of moderate-to-severe OHSS (OR 12.33; 95% CI 3.30–46.04), with consistently higher OHSS risk observed across hCG doses. Notably, no cases of OHSS were observed with the GnRHa-only trigger in either fresh or freeze-all cycles.

**Conclusion:**

In high ovarian responders, dual trigger with low-dose hCG does not confer a reproductive benefit over GnRHa-only trigger but is associated with a higher observed incidence of moderate-to-severe OHSS. Because the included studies were limited in number and heterogeneous in embryo-transfer strategy, ovarian stimulation protocols, and hCG dosing, these findings should be interpreted with caution and regarded as hypothesis-generating. In particular, as fresh transfer cycles modify OHSS risk and subgroup analysis was not feasible, the applicability of these results to contemporary universal freeze-all practice remains uncertain.

## Introduction

Ovarian hyperstimulation syndrome (OHSS) remains one of the most serious complications of assisted reproductive technology (ART), with the potential to result in significant morbidity and, in severe cases, life-threatening consequences [1, 2]. High ovarian responders, most of whom have PCOS, generally produce a high number of oocytes, yet tend to have lower oocyte maturity and a markedly increased risk of OHSS [3, 4]. OHSS is driven almost entirely by hCG exposure, which triggers granulosa cell luteinization, VEGF overproduction, and pathologic vascular permeability leading to ascites, hemoconcentration, and in severe cases, thromboembolic or respiratory complications [5].

GnRH agonist-only (GnRHa-only) trigger in antagonist cycles has therefore emerged as a preferred strategy for final oocyte maturation in high responders [6, 7]. However, the efficacy of the GnRHa-only trigger is hampered the short duration of the resultant luteinizing hormone (LH) surge and shorter half-life compared to hCG leads to early luteolysis and defective corpus luteal function, hence lower reproductive outcomes [8, 9]. In addition, GnRHa-only trigger has been associated with reduced oocyte yield and, in some reports, lower oocyte maturity compared with hCG-containing triggers [10]. The dual trigger, combining GnRHa with low-dose hCG, was developed to improve oocyte maturation and embryo quality by overcoming the shorter half-life of GnRHa, thereby resulting in a better reproductive outcome [11, 12]. While some studies suggested better oocyte maturity or pregnancy rates, more recent findings show inconsistent effects on live birth, and growing concern that even low-dose hCG reintroduces measurable OHSS risk in high responders [13, 14]. However, the true magnitude of this risk remains uncertain, as some studies have reported no OHSS events despite hCG exposure [11].

Because high responders represent the group most vulnerable to OHSS and most likely to produce large numbers of oocytes, determining whether a dual with low-dose hCG trigger provides true clinical benefit without unacceptable risk remains clinically important. To evaluate whether dual trigger improves reproductive outcomes as oocyte retrieved, mature oocytes, top-quality embryos, fertilization rate, CPR, LBR, and CLBR compared with GnRHa-only trigger in high ovarian responders undergoing GnRH antagonist IVF/ICSI cycles, and to quantify the associated risk of miscarriage rate and moderate-to-severe OHSS.

## Material and Methods

### Search Strategy and Selection Criteria

This systematic review and meta-analysis were conducted in accordance with the PRISMA guidelines [15]. The completed PRISMA 2020 checklist is provided as online supplementary Material (for all online suppl. material, see https://doi.org/10.1159/goi/aczag007) (Supplementary Table S2). A comprehensive literature search of PubMed, Scopus, and Google Scholar was conducted from inception to December 2025, supplemented by manual screening of the reference lists of eligible studies and relevant reviews. The search strategy combined controlled vocabulary and free-text terms related to ovulation trigger and ovarian response, including gonadotropin-releasing hormone agonist, dual trigger, low-dose hCG, high ovarian responder, in vitro fertilization, and OHSS. Eligible studies included randomized controlled trials or observational cohort studies involving high ovarian responders undergoing IVF/ICSI that compared dual trigger (GnRHa plus low-dose hCG) with GnRHa-only trigger and reported at least one reproductive outcome or moderate-to-severe OHSS. Studies using hCG-only triggers, mixed responder populations without stratified analysis, lacking a comparator group, or reporting duplicate data were excluded.

### Extraction and Quality Assessment

Three investigators (H.H.S., M.A.R., and N.A.) independently screened titles, abstracts, and full texts. Disagreements were resolved by consensus. Data were extracted independently including study design, patient characteristics and definition of high response, trigger protocols, hCG dosing, reproductive outcomes, and incidence of moderate-severe OHSS. Randomized trials were assessed using the Cochrane Risk of Bias 2.0 tool, and non-randomized studies were evaluated using ROBINS-I [16, 17]. Assessments were performed independently by three reviewers (H.H.S., M.A.R., and N.A.). Any discrepancies were resolved through consensus or adjudication by a third author (H.B.). The primary outcomes were the number of oocytes retrieved, mature oocyte rate, fertilization rate, proportion of top-quality embryos, and incidence of moderate-to-severe OHSS. The secondary outcomes included clinical pregnancy rate (CPR), live birth rate (LBR), cumulative live birth rate (CLBR), and miscarriage rate.

### Data Synthesis and Statistical Analysis

For reproductive outcomes, pooled effects were calculated using risk ratios (RRs) with 95% confidence intervals (CIs). Due to the low incidence and frequent zero-event studies, odds ratios (ORs) were used for OHSS outcomes. A random-effects model (Mantel-Haenszel method) was applied as the primary approach. Statistical heterogeneity was assessed using the I^2^ statistic, with values >50% indicating substantial heterogeneity. Sensitivity analyses were conducted by excluding studies at high risk of bias and by comparing fixed- and random-effects models. All analyses were performed using Review Manager (RevMan) version 5.4 and MedCalc (v19.5.1) [18, 19]. Evidence certainty was evaluated according to the GRADE framework, applying established criteria for rating confidence in effect estimates. The assessment process was supported by the GRADEpro Guideline Development Tool (McMaster University, Evidence Prime Inc.), available at https://GRADEpro.org [20]. This review has been registered with PROSPERO (CRD420251252178).

## Results

The electronic database search identified 425 records. After removal of duplicates and screening of titles and abstracts, potentially relevant studies underwent full-text assessment. Following full-text review, 65 studies were excluded for reasons including inappropriate population, inappropriate intervention or trigger protocol, absence of a comparator group, mixed responder populations without stratified analysis, duplicate datasets, or insufficient outcome reporting. Finally, five retrospective cohort studies met the inclusion criteria and were included in the final analysis (Fig. 1). All of the included studies comprise high ovarian responders undergoing IVF/ICSI with a GnRH antagonist protocol [10–14]. Studies were conducted in Taiwan, China, and the USA. The mean age of participants across studies ranged from approximately 29–33 years, with no clinically meaningful differences between trigger groups within individual cohorts. Definitions of high response varied but consistently reflected increased OHSS risk, including PCOS or polycystic ovarian morphology (PCOM), prior OHSS, high follicle counts (≥13–15 follicles), elevated estradiol levels on trigger day, or ≥15 oocytes retrieved. All studies compared a GnRH agonist-only trigger (triptorelin or leuprolide acetate) with a dual trigger combining the same GnRH-agonist and low-dose hCG (500–2,000 IU), with one study further stratifying by hCG dose (Tables 1, 2). All included studies were assessed as having a moderate risk of bias (Fig. 2). The detailed electronic search strategy used for study identification across all databases is summarized in supplementary table S1.

**Fig. 1.**
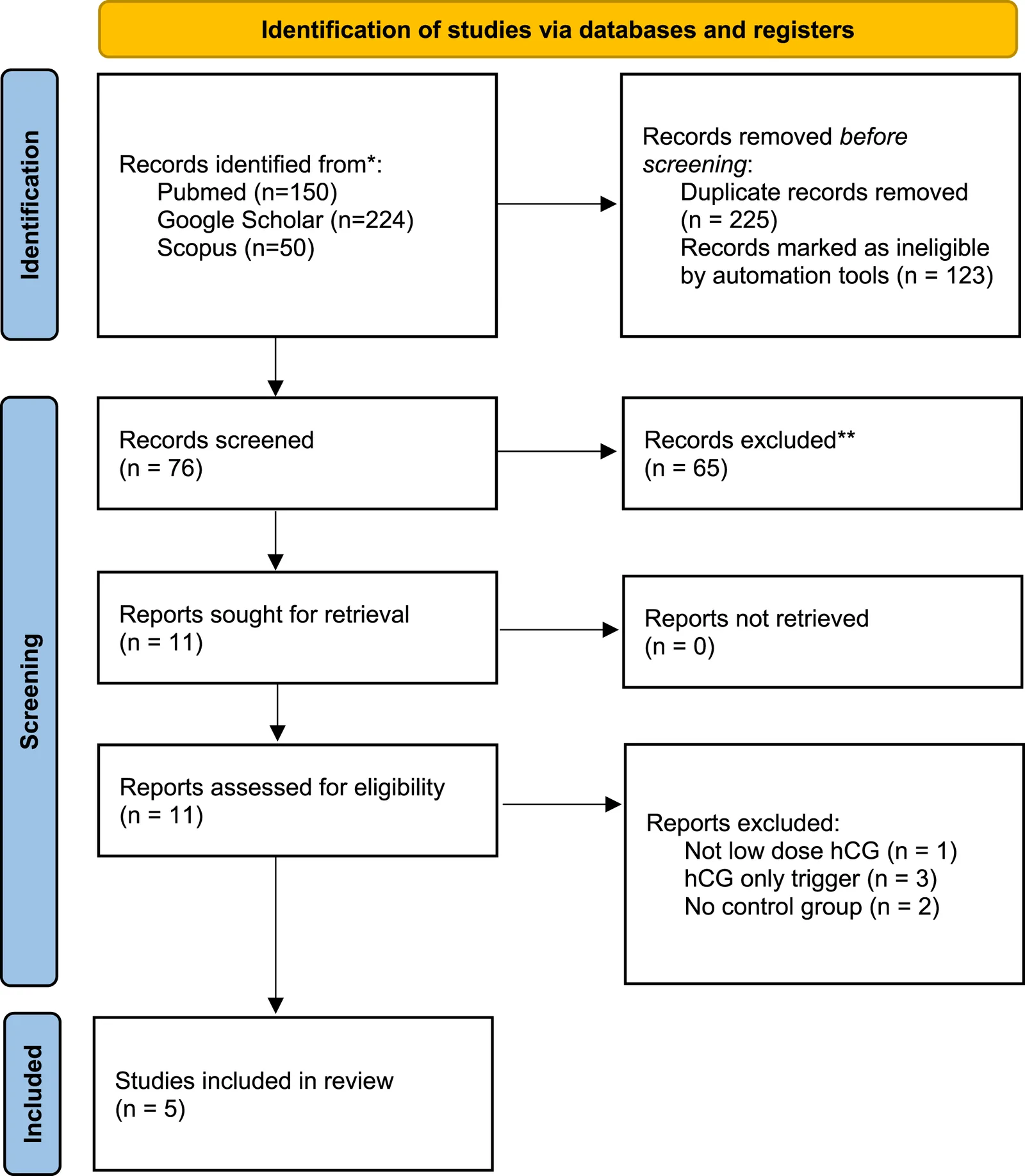
PRISMA flow diagram illustrating the literature search and study selection process for this meta-analysis [15].

**Table 1.** Characteristics of included studies

No	Author	Country	Study design	High responder definition	Trigger		Protocol	Embryo transfer
1	Huang et al., 2016 [11]	Taiwan	Retrospective study	PCOM PCOS Patients with a history of OHSS Patients with peak estradiol (E2) ≥ 2,500 pg/mL on trigger day	GnRHa trigger only	Triptorelin 0.2 mg SC (No hCG given)	Antagonist protocol	Fresh transfer
					Dual Trigger	Triptorelin 0.2 mg + low-dose hCG (500–1000 IU) based on E2 and follicle number	Antagonist protocol	
2	Griffin et al., 2012 [12]	USA	Retrospective study	Previous OHSS, OR Previous cycle canceled due to OHSS risk, OR ≥13 follicles ≥11 mm, OR Peak E2 < 4,000 pg/mL but still high responders per follicle count	GnRH only	Leuprolide acetate 1 mg SC	Antagonist protocol	Fresh transfer
					Dual trigger	Leuprolide acetate 1 mg SC + 1000 IU hCG (fixed dose)		
3	O’Neill et al., 2016 [10]	USA	Retrospective study	Prior cycle history Age Ovarian reserve Infertility diagnosis PCOS (Rotterdam) High AFC High E2 Large number of follicles on the day of trigger	GnRH only	Leuprolide acetate 4 mg only	Antagonist protocol	Fresh transfer
						No hCG exposure		
					Dual trigger	Leuprolide acetate 4 mg (80 units) + 1000 IU hCG		
4	He et al., 2022 [14]	China	Retrospective study	Retrieved oocytes ≥ 15	GnRH only	Triptorelin 0.2 mg	Antagonist protocol	Freeze-all strategy
					Dual trigger (low-dose hCG)	Triptorelin 0.2 mg + 1000 IU hCG		
					Dual trigger (higher hCG)	Triptorelin 0.2 mg + 2000 IU hCG		
5	Binbin et al., 2023 [13]	China	Retrospective study	Peak E2 on trigger day > 14,640 pmol/L, and/or Number of oocytes retrieved > 15	GnRH only	Triptorelin 0.2 mg	Antagonist Protocol	Freeze-all strategy
					Dual trigger	Triptorelin 0.2 mg combined with low-dose hCG (2,000 IU)		

*E2, estradiol; OHSS, ovarian hyperstimulation syndrome; hCG, human chorionic gonadotropin; SC, subcutaneous; AFC, antral follicle count; PCOM, polycystic ovarian morphology; PCOS, polycystic ovarian syndrome; GnRHa, gonadotropin-releasing hormone agonist.

**Table 2.** Baseline characteristics of women undergoing IVF across included studies

No	Author	Trigger	Age	Parity	BMI (kg/m^2^)	Baseline AMH	AFC	Maximum E2 (pg/mL)	E2 on trigger day (pg/mL)	Follicles on trigger day
1	Huang et al., 2016 [11]	GnRHa-only (*n* = 23)	32.6±3.1	0	22.0±3.0	–	–	–	4,663±1,545	20.1±5.7
				20/23 (87%)						
				≥1						
				3/23 (13%)						
		Dual trigger (*n* = 33)	33.5±4.1	0	22.1±4.3	–	–	–	5,390±2,212	22.7±8.0
				26/33 (78.8%)						
				≥1:7/33 (21.2%)						
2	Griffin et al., 2012 [12]	GnRHa-only (*n* = 68)	31.9±3.9	0.3±0.6	26.8±6.0	–	–	2,814±779	–	–
		Dual trigger (*n* = 34)	31.5±3.8	0.3±0.4	24.4±5.3	–	–	2,568±748	–	–
3	O’Neill et al., 2016 [10]	GnRHa-only (*n* = 108)	32.4 (30.3–35.1)	–	23 (21–27)	–	23 (16–31)	4,360 (3517–5,704)	3604 (2,843–4,536)	31 (25–35)
		Dual trigger (*n* = 66)	31.0 (29.0–34.1)	–	23 (20.7–25.3)	–	18 (11–25)	3541 (2,943–5,014)	2,993 (2,597–4,280)	28 (22–34)
4	He et al., 2022 [14]	GnRHa-only (*n* = 577)	29.63±3.61	–	21.92±3.54	9.70±3.76	–	–	16,896.28±3052.42 pmol/L	–
		Dual trigger (low dose hCG) (*n* = 403)	29.78±3.65	–	22.16±3.64	9.52±4.11	–	–	16,797.20±2,944.31 pmol/L	–
		Dual trigger (higher hCG) (*n* = 363)	30.06±3.79	–	22.39±3.47	9.31±4.40	–	–	16,725.54±3094.0 pmol/L	–
5	Binbin et al., 2023 [13]	GnRHa-only (*n* = 251)	30.68±3.64	0 (0–1)	22.73±2.89	8.89±4.78	19.30±6.32	–	22,905.84±9,513.28 pmol/L	–
		Dual trigger (*n* = 741)	30.38±3.41	0 (0–1)	22.43±2.82	8.17±4.69	18.58±5.55	–	17,200.82±6,811.64 pmol/L	–

*BMI, body mass index; AMH, anti-Müllerian hormone; AFC, antral follicle count; E2, estradiol.

**Fig. 2.**
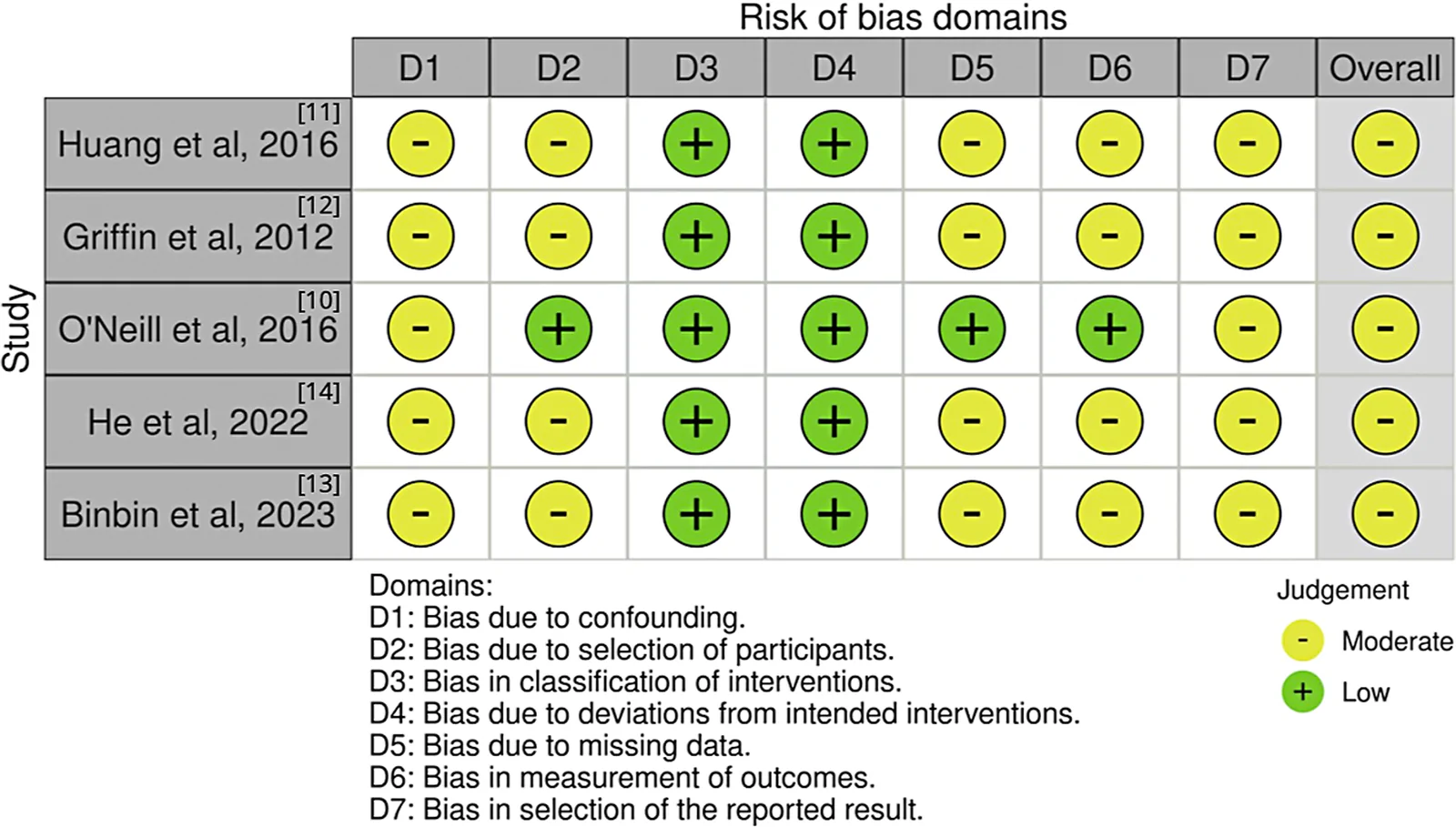
Risk of bias assessment conducted using the ROBINS-I tool for non-randomized studies [16].

### Oocyte Retrieved

There was no significant difference in the number of oocytes retrieved between dual trigger and GnRHa-only trigger (MD −0.19, 95% CI −1.01 to 0.64; *p* = 0.66; I^2^ = 44%), as shown in Figure 3a, Table 3. Subgroup analyses by hCG dose demonstrated similarly nonsignificant differences for both 1,000 IU hCG (MD 0.74, 95% CI −1.14 to 2.62) and 2,000 IU hCG (MD −0.64, 95% CI −1.73 to 0.45), with no evidence of a subgroup effect (*p* = 0.21).

**Fig. 3.**
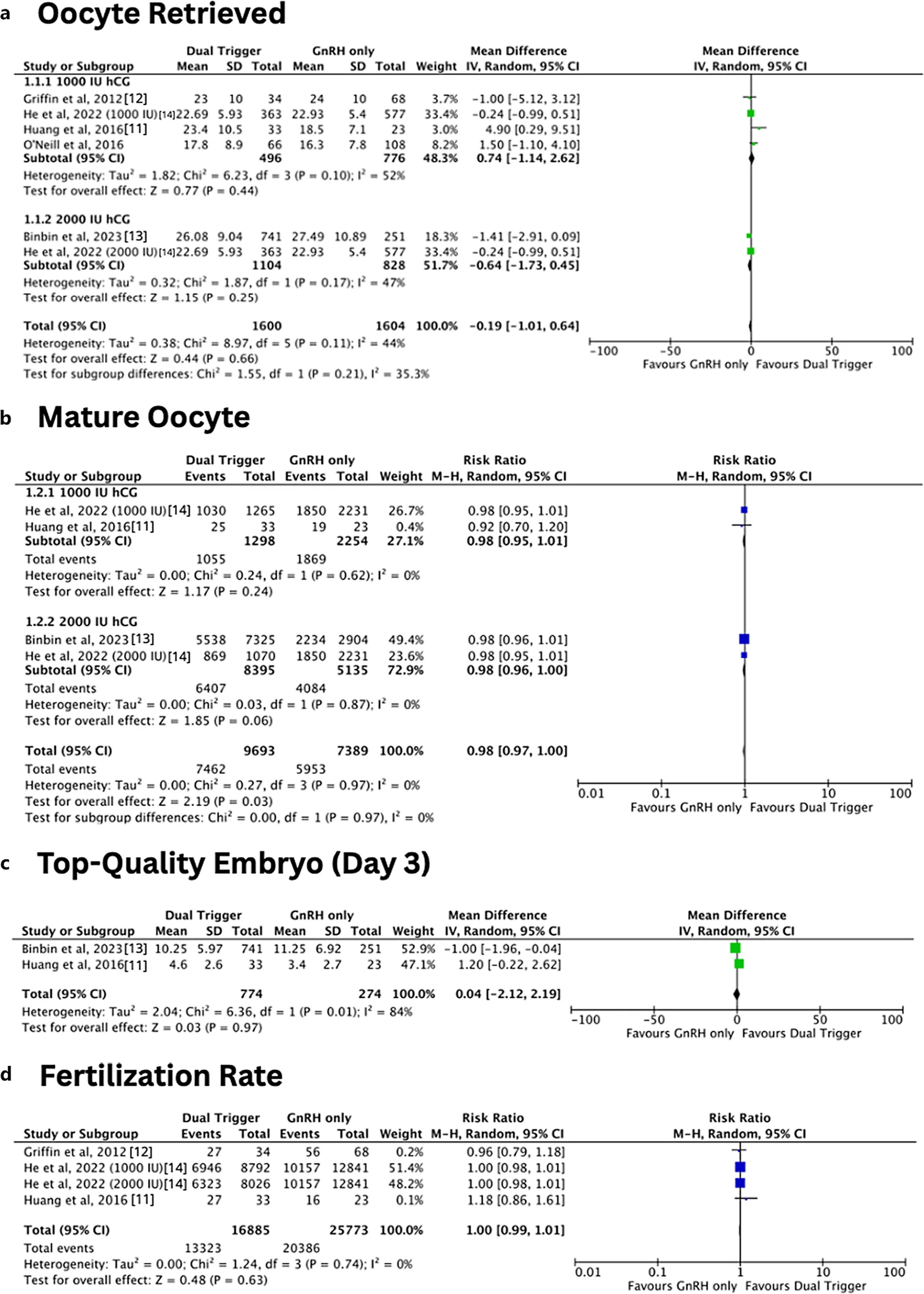
Forest plots comparing embryological outcomes between dual trigger and GnRH agonist-only trigger in high responders undergoing GnRH antagonist cycles. **a** Number of oocytes retrieved, mature (MII) oocytes (**b**), top-quality embryos on day 3 (**c**), and fertilization rate (**d**).

**Table 3.** Embryological and IVF outcomes

No	Author	Trigger	Oocyte retrieved	Mature oocyte (%)	TQE (day-3) (%)	Fertilization rate (%)	Implantation rate (%)	CPR (%)	LBR (%)	CLBR (%)	Miscarriage rate (%)	Moderate-Severe OHSS (%)
1	Huang et al., 2016 [11]	GnRHa-only (*n* = 23)	18.5±7.1	80.8±18.5	3.4±2.7	68.5±20.7	5.8±2.8	4/23 (17.24)	–	–	4/23 (50.0)	0/23 (0)
		Dual trigger (*n* = 33)	23.4±10.5	74.6±18.1	4.6±2.6	82.2±10.0	22.5±5.2	16/23 (48.5)	–	–	3/33 (16.7)	0/33 (0)
2	Griffin et al., 2012 [12]	GnRHa-only (*n* = 68)	24±10	–	–	81.9±18.1	81.9±18.1	25/68 (36.8)	21/68 (30.9)	–	6/43 (14.0)	0/68 (0)
		Dual trigger (*n* = 34)	23±10	–	–	79.2±13.9	79.2±13.9	20/34 (58.8)	18/34 (52.9)	–	3/26 (11.5)	1/34 (2.9)
3	O’Neill et al., 2016 [10]	GnRHa-only (*n* = 108)	16.5 (11–21.5)	70 (56–85)	–	–	–	68/108 (63)	–	​	6/108 (6)	0/108 (0)
		Dual trigger (*n* = 66)	17.5 (12–24)	82 (74–91)	–	–	–	29/66 (44)	–	​	5/66 (7)	4/66 (6)
4	He et al., 2022 [14]	GnRHa-only (*n* = 577)	22.93±5.40	1,850/2,231 (82.92)	2,036/10,017 (20.33)	10,157/12,841 (79.10)	–	364/557 (65.35)	302/557 (54.22)	429/577 (74.35)	37/364 (10.16)	0/577 (0)
		Dual trigger (low dose hCG) (*n* = 403)	22.41±5.25	1,030/1,265 (81.42)	1,416/6,823 (20.75)	6,946/8,792 (79.0)	–	247/389 (63.05)	212/389 (54.50)	305/403 (75.68)	23/247 (9.31)	6/403 (1.49)
		Dual trigger (higher hCG) (*n* = 363)	22.69±5.93	869/1,070 (81.21)	1,249/6,227 (20.06)	6,323/8,026 (78.78)	–	232/352 (65.91)	191/352 (54.26)	253/363 (69.70)	28/232 (12.07)	5/363 (1.38)
5	Binbin et al., 2023 [13]	GnRHa-only (*n* = 251)	27.49±10.89	2,234/2,904 (76.9)	11.25±6.92	–	–	175/364 (48.1)	144/364 (39.6)	142/247 (57.5)	144/364 (39.6)	0/251 (0)
		Dual trigger (*n* = 741)	26.08±9.04	5,538/7,325 (75.6)	10.25±5.97	–	–	552/1,176 (46.9)	448/1,176 (38.1)	446/731 (51.0)	448/1,176 (38.1)	11/741 (1.5)

*CPR clinical pregnancy rate; OHSS, ovarian hyperstimulation syndrome; CLBR, cumulative live birth rate; LBR, live birth rate; TQE, top-quality embryo.

### Mature Oocytes

There was no clinically meaningful difference in the mature oocyte rate between dual trigger and GnRHa-only trigger. The pooled analysis showed a RR of 0.98 (95% CI; 0.97–1.00; *p* = 0.03), with no heterogeneity (I^2^ = 0%), and subgroup analyses by hCG dose (1,000 IU vs. 2,000 IU) demonstrated consistent results with no subgroup effect (*p* = 0.97) (Fig. 3b, Table 3).

### Top-Quality Embryo (Day 3)

There was no significant difference in the number of day-3 total quality embryos between dual trigger and GnRHa-only trigger (MD 0.04, 95% CI; −2.12 to 2.19; *p* = 0.97), although substantial heterogeneity was observed (I^2^ = 84%) (Fig. 3c, Table 3).

### Fertilization Rate

There was no significant difference in the number of fertilization rate between dual trigger and GnRHa-only trigger (RR 1.00, 95% CI; 0.99 to 1.01; *p* = 0.63), with no heterogeneity (I^2^ = 0%) (Fig. 3d, Table 3).

### Clinical Pregnancy Rate

There was no significant difference in clinical pregnancy rate between dual trigger and GnRHa-only trigger (RR 1.01, 95% CI; 0.88–1.17; *p* = 0.86), with substantial heterogeneity across studies (I^2^ = 74%). Subgroup analyses by hCG dose (1,000 IU vs. 2,000 IU) showed no significant differences and no evidence of a subgroup effect (*p* = 0.39) (Fig. 4a, Table 3).

**Fig. 4.**
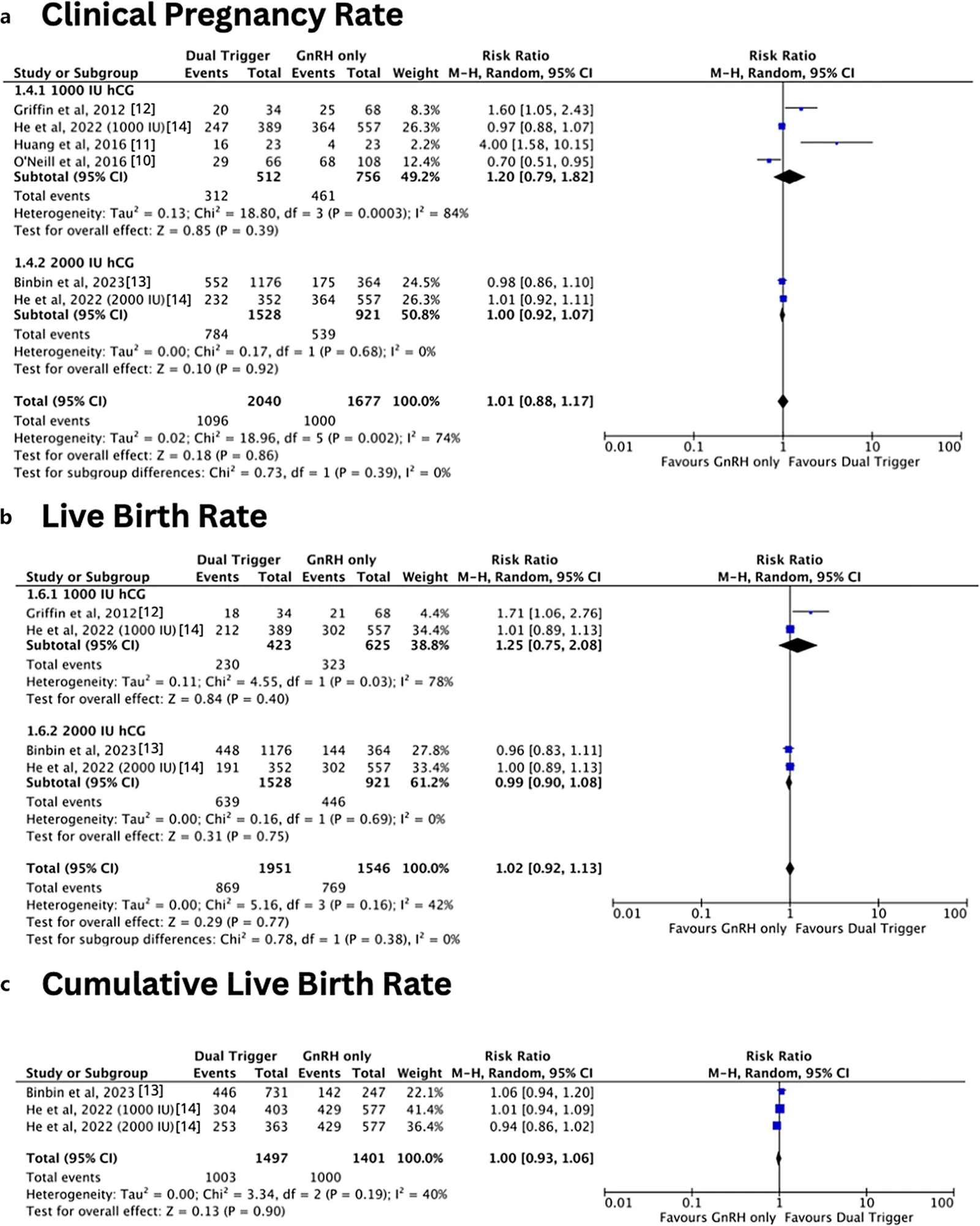
Forest plots comparing pregnancy outcomes between dual trigger and GnRH agonist-only trigger in high responders undergoing GnRH antagonist cycles. **a** Clinical pregnancy rate, (**b**) live birth rate, and (**c**) cumulative live birth rate.

### Live Birth Rate and Cumulative Live Birth Rate

There was no significant difference in live birth rate between dual trigger and GnRHa-only trigger (RR 1.02, 95% CI; 0.92–1.13; *p* = 0.77; I^2^ = 42%) (Fig. 4b, Table 3). Subgroup analyses by hCG dose (1,000 IU vs. 2,000 IU) showed no significant differences and no subgroup effect (*p* = 0.38). There was no significant difference in CLBR between dual trigger and GnRHa-only trigger (RR 1.00, 95% CI; 0.93–1.06; *p* = 0.90; I^2^ = 40%) (Fig. 4c, Table 3).

### Miscarriage Rate

Across the included studies, miscarriage rates were comparable between the dual-trigger and GnRH agonist-only trigger groups. Pooled analysis demonstrated no statistically significant difference in miscarriage risk (RR = 0.97, 95% CI 0.85–1.11; *p* = 0.69). Subgroup analyses by hCG dose (1,000 IU vs. 2,000 IU) showed no significant differences and no subgroup effect (*p* = 0.73) (Fig. 5a, Table 3).

**Fig. 5.**
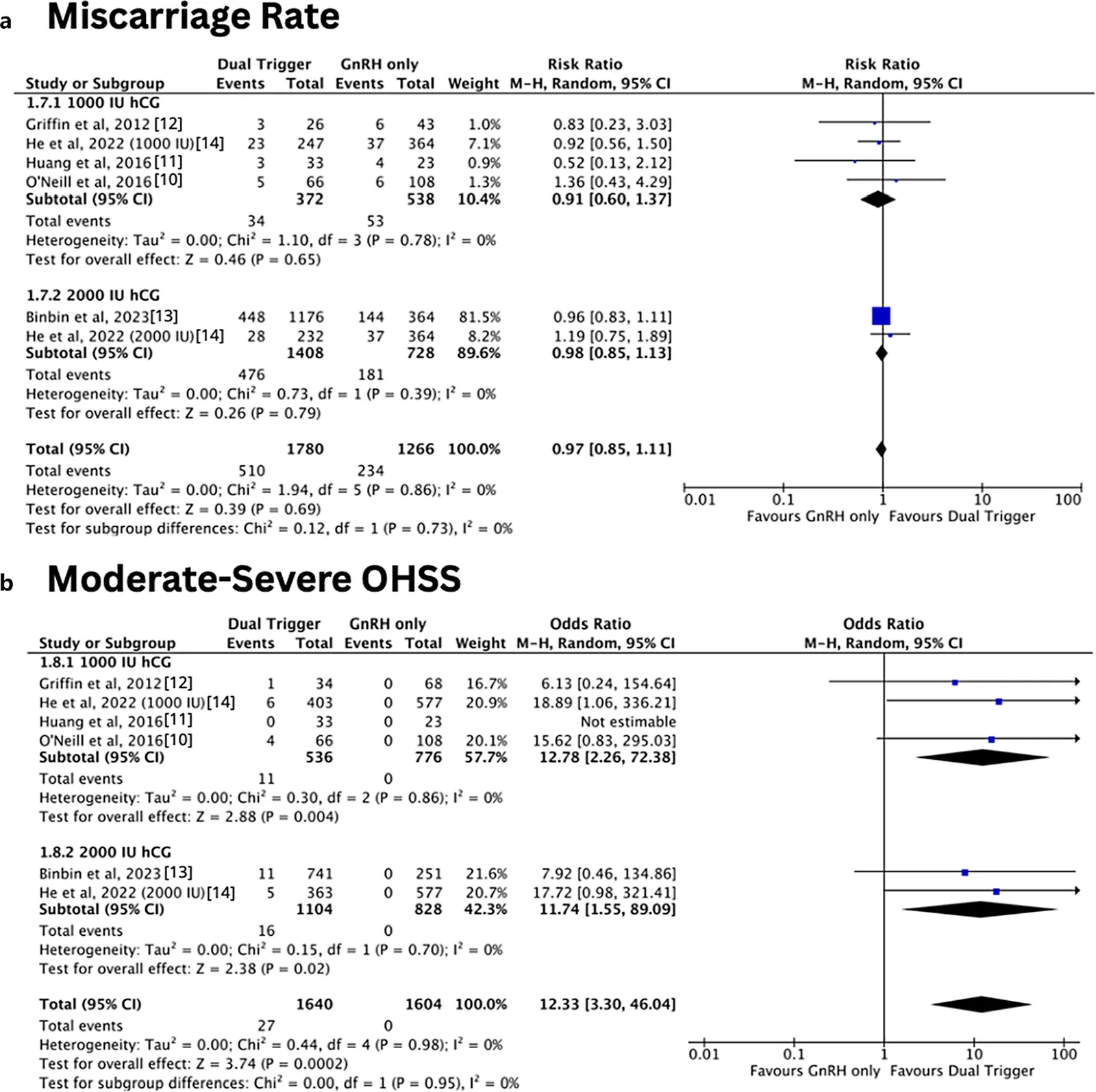
Forest plots of safety outcomes comparing dual trigger and GnRH agonist-only trigger in high responders. **a** Miscarriage rate and moderate-to-severe OHSS (**b**).

### Moderate-Severe OHSS

In this review, dual trigger was associated with a higher occurrence of moderate-to-severe OHSS compared with the GnRHa-only trigger (OR 12.33, 95% CI: 3.30–46.04; *p* = 0.0002; I^2^ = 0%) (Fig. 5b, Table 3). Subgroup analyses demonstrated consistently increased odds of OHSS with both 1,000 IU hCG (OR 12.78, 95% CI: 2.26–72.38) and 2,000 IU hCG (OR 11.74, 95% CI: 1.55–89.09). Proportional meta-analysis showed a pooled incidence of moderate-to-severe OHSS of 1.8% (95% CI: 1.00–2.99; I^2^ = 32.85%) in the dual trigger group (Fig. 6a). In comparison, the GnRHa-only trigger group demonstrated a pooled OHSS incidence of 0%, as no cases of OHSS were reported across the included studies (Fig. 6b, Table 3).

**Fig. 6.**
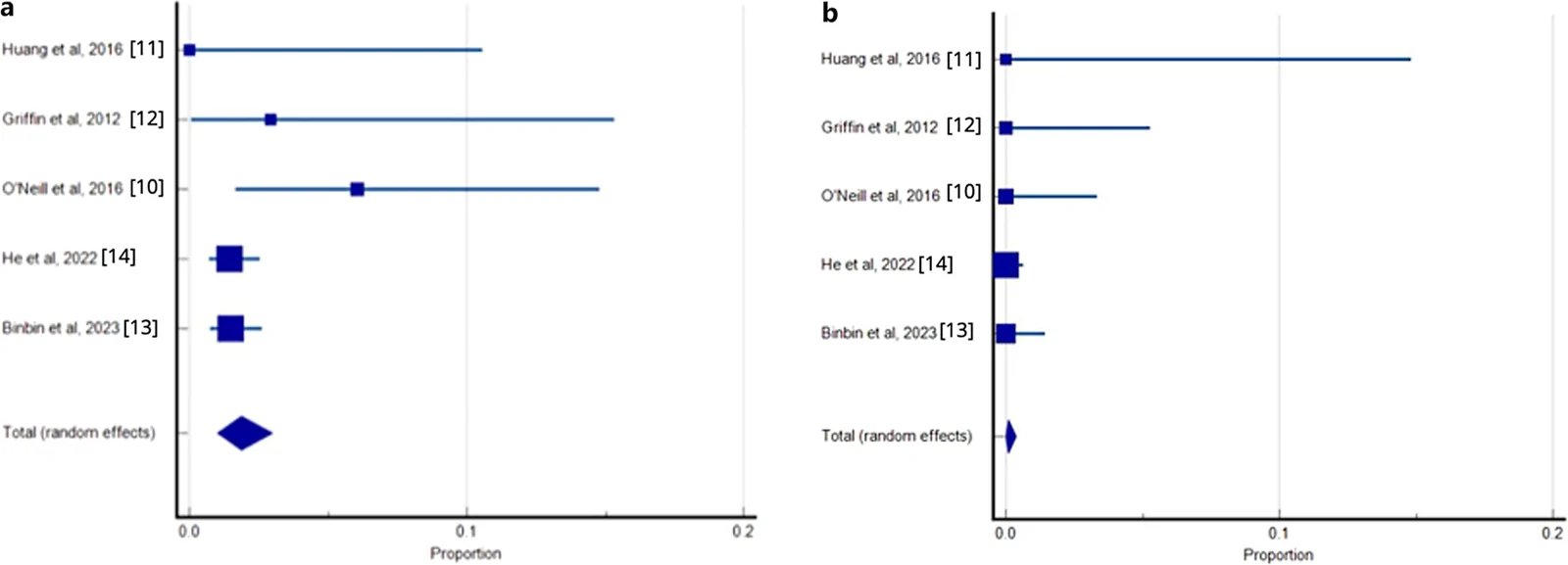
Proportional meta-analysis illustrating the pooled incidence of moderate-to-severe ovarian hyperstimulation syndrome (OHSS) in high responders undergoing dual trigger (**a**) and GnRH agonist-only trigger (**b**) protocol.

### Confidence in Cumulative Evidence

The certainty of evidence was assessed using the GRADE approach and is summarized in Table 4. As all included studies were non-randomized cohort studies, the certainty of evidence for all outcomes initially started at low. For laboratory outcomes, including the number of oocytes retrieved, mature (MII) oocytes, top-quality embryos, and fertilization rate, the certainty of evidence was rated as moderate, downgraded by one level for serious risk of bias due to the observational study design and non-random allocation of trigger strategy. No serious concerns were identified regarding inconsistency, indirectness, or imprecision. For reproductive outcomes, including clinical pregnancy rate, live birth rate, and cumulative live birth rate, the certainty of evidence was also rated as moderate, downgraded by one level for serious risk of bias related to potential confounding. Consistency of effect estimates and adequate precision precluded further downgrading. The certainty of evidence for miscarriage rate was rated as moderate, downgraded for risk of bias, with no additional concerns regarding inconsistency, indirectness, or imprecision. For moderate-to-severe OHSS, the certainty of evidence was rated as low. The evidence was downgraded by one level for serious risk of bias due to the observational study design and potential confounding, and by an additional level for imprecision owing to the small number of events and the presence of zero-event cells in several studies, which resulted in wide confidence intervals. Although the direction of effect was consistent across studies, the limited number of events reduces the precision of the effect estimate. Overall, the certainty of evidence ranged from low to moderate across outcomes, with risk of bias related to the observational study design representing the primary limitation. No outcome met criteria for high-certainty evidence under the GRADE framework.

**Table 4.** GRADE evidence profile of primary and secondary outcomes

Certainty assessment							Patients, *n*		Effect		Certainty	Importance
studies, *n*	study design	risk of bias	inconsistency	indirectness	imprecision	other considerations	dual trigger with low-dose HCG	GnRHa only trigger	relative (95% ci)	absolute (95% CI)		
*Oocyte Retrieved*												
5	Non-randomized studies	Serious^a^	Not serious	Not serious	Not serious	None	1,640	1,027	–	MD **0.19 lower**	⨁⨁⨁◯	IMPORTANT
										(1.01 lower to 0.64 higher)	Moderate^a^	
*Mature Oocyte*												
3	Non-randomized studies	Serious^a^	Not serious	Not serious	Not serious	None	7,462/9,693 (77.0%)	5,953/7,389 (80.6%)	**RR 0.98**	**16 fewer** per **1,000**	⨁⨁⨁◯	IMPORTANT
									(0.97–1.00)	(from 24 fewer to 0 fewer)	Moderate^a^	
*Top-Quality Embryo*												
2	Non-randomized studies	Serious^a^	Serious^b^	Not serious	Not serious	None	774	274	–	MD **0.04 higher**	⨁⨁◯◯	IMPORTANT
										(2.12 lower to 2.19 higher)	Low^a^^,^^b^	
*Fertilization Rate*												
3	Non-randomized studies	Serious^a^	Not serious	Not serious	Not serious	None	13,323/16,885 (78.9%)	20,386/25,773 (79.1%)	**RR 1.00**	**0 fewer** per **1,000**	⨁⨁⨁◯	IMPORTANT
									(0.99–1.01)	(from 8 fewer to 8 more)	Moderate^a^	
*Clinical Pregnancy Rate*												
5	Non-randomized studies	Serious^a^	Serious^b^	Not serious	Not serious	None	1,096/2,040 (53.7%)	1,000/1,677 (59.6%)	**RR 1.01**	**6 more** per **1,000**	⨁⨁◯◯	IMPORTANT
									(0.88–1.17)	(from 72 fewer to 101 more)	Low^a^^,^^b^	
*Live Birth Rate*												
3	Non-randomized studies	Serious^a^	Not serious	Not serious	Not serious	None	869/1,951 (44.5%)	769/1,546 (49.7%)	**RR 1.02**	**10 more** per **1,000**	⨁⨁⨁◯	IMPORTANT
									(0.92–1.13)	(from 40 fewer to 65 more)	Moderate^a^	
*Cumulative Live Birth Rate*												
2	Non-randomized studies	Serious^a^	Not serious	Not serious	Not serious	None	1,003/1,497 (67.0%)	1,000/1,401 (71.4%)	**RR 1.00**	**0 fewer** per **1,000**	⨁⨁⨁◯	IMPORTANT
									(0.93–1.06)	(from 50 fewer to 43 more)	Moderate^a^	
*Miscarriage Rate*												
5	Non-randomized studies	Serious^a^	Not serious	Not serious	Not serious	None	510/1,780 (28.7%)	0/1,266 (0.0%)	**RR 0.97**	**0 fewer** per **1,000**	⨁⨁⨁◯	IMPORTANT
									(0.85–1.11)	(from 0 fewer to 0 fewer)	Moderate^a^	
*Moderate-severe OHSS*												
5	Non-ndomized studies	Serious^a^	Not serious	Not serious	Serious^c^	None	27/1,640 (1.6%)	0/1,604 (0.0%)	**OR 12.33**	**0 fewer** per **1,000**	⨁⨁◯◯	CRITICAL
									(3.30–46.04)	(from 0 fewer to 0 fewer)	Low^a^^,^^c^	

CI, confidence interval; MD, mean difference; OR, odds ratio; RR, risk ratio.

^a^ Non-randomized studies and allocation to trigger strategy was based on clinician preference or institutional protocol.

^b^ Inconsistency owing to substantial statistical heterogeneity.

^c^ Imprecision was downgraded for OHSS due to the small number of events and wide confidence intervals despite consistent direction of effect.

## Discussion

This meta-analysis demonstrates that, in high ovarian responders undergoing GnRH antagonist cycles, dual trigger does not improve reproductive outcomes compared with GnRHa-only trigger, including oocyte yield, mature oocyte rate, fertilization, embryo quality, clinical pregnancy, live birth, and cumulative live birth rates. In contrast, dual trigger is consistently associated with a higher risk of moderate-to-severe OHSS. These findings suggest that the routine addition of hCG in high responders provides no measurable reproductive benefit but introduces avoidable maternal risk.

The consistency of these neutral findings across laboratory and clinical outcomes supports the concept that GnRHa-only trigger is sufficient to induce final oocyte maturation in high responders [14]. The endogenous LH and FSH surge elicited by GnRHa appears adequate to support oocyte competence, embryo development, and subsequent implantation [21]. These findings are further supported by studies employing freeze-all strategies, which report comparable cumulative live birth rates in line with similar oocyte retrieval efficiency using GnRHa-only triggering [22].

In the absence of clear efficacy differences between strategies, attention naturally shifts toward differences in safety profiles. The most clinically consequential finding of this review is the markedly increased risk of moderate-to-severe OHSS associated with dual trigger. Despite the use of low-dose hCG, the odds of OHSS were more than tenfold higher compared with GnRHa-only trigger. Prolonged luteotropic exposure from exogenous hCG increases OHSS risk because of its longer half-life compared with the endogenous LH surge induced by GnRHa [23, 24]. O’Neill et al. demonstrated a clear increase in severe OHSS with dual trigger compared with GnRHa alone, despite modest gains in oocyte yield [10]. Importantly all the cohorts included report near-zero OHSS incidence with GnRHa-only trigger, emphasizing its role as one of the most effective preventive strategies currently available [10–14]. Critically, this absence of moderate-to-severe OHSS in the GnRHa-only arm was observed uniformly across studies regardless of embryo-transfer strategy, with no cases reported in either fresh-transfer or freeze-all cohorts, indicating that the safety advantage is intrinsic to the trigger itself and not an artefact of transfer strategy.

Viewed from a risk-benefit perspective, any theoretical advantage of hCG supplementation is outweighed by its disproportionate contribution to OHSS. This concern is particularly relevant in fresh embryo-transfer cycles, where continued luteal and early pregnancy exposure to hCG may further amplify OHSS risk, but it also extends to patients managed with planned freeze-all strategies [25, 26]. Taken together, these findings support GnRHa-only trigger as the preferred default approach for high responders. Dual trigger should therefore not be used routinely in high responder and, if considered, should be limited to carefully selected cases with prior documented suboptimal oocyte maturation following GnRHa-only trigger, with explicit recognition of the associated OHSS risk.

Several limitations should be acknowledged. The number of eligible studies was limited, and although randomized controlled trials were eligible for inclusion, none meeting the inclusion criteria were identified; all included studies were retrospective in design, introducing potential selection bias and residual confounding inherent to observational data. Definitions of high ovarian response varied slightly across studies, although heterogeneity for OHSS outcomes remained low. In addition, several included studies were conducted predominantly in fresh embryo-transfer settings (Griffin et al. [12] O’Neill et al. [10] and Huang et al. [11]), while others employed freeze-all strategies (Binbin et al. [13] and He et al. [14]). Notably, no moderate-to-severe OHSS occurred in the GnRHa-only arm of any study in either setting, indicating that the observed safety advantage was consistent across both fresh and freeze-all transfer strategies and is unlikely to be an artefact of transfer strategy. Because fresh transfer entails luteal-phase and early pregnancy exposure to hCG, which may increase OHSS risk, the generalizability of these findings to contemporary freeze-all practice may be limited. Due to the small number of studies reporting outcomes stratified by transfer type and heterogeneity in protocols, subgroup analysis based on embryo-transfer strategy was not feasible, the pooled estimates should therefore be regarded as hypothesis-generating rather than definitive. Furthermore, the meta-analysis relied primarily on crude estimates reported in the original publications, as adjusted effect estimates and detailed baseline characteristics (e.g., age, anti-Müllerian hormone levels, antral follicle count, estradiol levels, and polycystic ovary syndrome status) were not consistently available across studies, precluding adjustment for potential confounders. Therefore, confounding by indication related to baseline OHSS risk cannot be excluded.

Future studies should prioritize prospective, ideally randomized, designs focusing on high-responder populations and contemporary freeze-all strategies. Standardized definitions of high ovarian response and OHSS, along with uniform trigger and luteal support protocols, would improve comparability across studies. In particular, adequately powered studies conducted exclusively in freeze-all cycles are needed to better delineate the true OHSS risk associated with hCG-containing triggers and to clarify their impact on oocyte and embryo outcomes independent of luteal-phase and early pregnancy effects.

## Conclusion

In high ovarian responders, dual trigger does not improve IVF outcomes compared with GnRHa-only triggering. However, it is associated with increased risk of moderate-to-severe OHSS. Collectively, these findings indicate that GnRHa-only trigger provides comparable efficacy with a superior safety profile. Avoidance of hCG exposure was associated with reduced OHSS incidence among high ovarian responders.

## Acknowledgments

This publication charge is funded by Unpad through the Indonesian Endowment Fund for Education (LPDP) on behalf of the Indonesian Ministry of Higher Education, Science and Technology and managed under the EQUITY Program (Contract No. 4303/B3/DT.03.08/2025 and 3927/UN6. RKT/HK.07.00/2025).

## Statement of Ethics

A statement of ethics is not applicable because this study is based exclusively on published literature.

## Conflict of Interest Statement

The author declares that there are no conflicts of interest.

## Funding Sources

This study has no funding support.

## Author Contributions

Hanom Husni Syam: writing – review and editing, writing – original draft, software, project administration, methodology, formal analysis, data curation, and conceptualization. Hartanto Bayuaji: writing – review and editing, writing – original draft, formal analysis, data curation, and conceptualization. Dian Tjahyadi: writing – review and editing, writing – original draft, formal analysis, data curation, and conceptualization. Anita Rachmawati: writing – review and editing, writing – original draft, formal analysis, data curation, and conceptualization. Mulyanusa Amarullah Ritonga: writing – review and editing, writing – original draft, formal analysis, data curation, and conceptualization. Nicholas Adrianto: writing – review and editing, writing – original draft, formal analysis, data curation, and conceptualization.

## Data Availability

As this meta-analysis was based on data extracted from previously published studies, all analyzed data are publicly available. The authors did not have access to individual patient-level data. Researchers interested in obtaining such data should contact the corresponding authors of the original studies. All data generated or analyzed in this study are included within this published article. Further inquiries may be addressed to the corresponding author.
